# Insights on the Hydrodynamics of Chiari Malformation

**DOI:** 10.3390/jcm11185343

**Published:** 2022-09-12

**Authors:** Cyrille Capel, Pauline Padovani, Pierre-Henri Launois, Serge Metanbou, Olivier Balédent, Johann Peltier

**Affiliations:** 1 Department of Neurosurgery, Hospital University Center of Amiens-Picardie, 80054 Amiens, France; 2 Chimère UR 7516, Jules Verne University, 80000 Amiens, France; 3 Radiology Department, Hospital University Center of Amiens-Picardie, 80054 Amiens, France; 4 Image Processing Department, Hospital University Center of Amiens-Picardie, 80054 Amiens, France

**Keywords:** Chiari malformation type 1, CSF, hydrodynamic, phase-contrast MRI

## Abstract

Background: We propose that the appearance of a ptosis of the cerebellar tonsils and syringomyelia is linked to its own hemohydrodynamic mechanisms. We aimed to quantify cerebrospinal fluid (CSF) and cerebral blood flow to highlight how neurofluid is affected by Chiari malformations type 1(CMI) and its surgery. Methods: We retrospectively included 21 adult patients with CMI who underwent pre- and postoperative phase contrast MRI (PCMRI) during the period from 2001 to 2017. We analyzed intraventricular CSF, subarachnoid spaces CSF, blood, and tonsils pulsatilities. Results: In preoperative period, jugular venous drainage seems to be less preponderant in patients with syringomyelia than other patients (venous correction factor: 1.49 ± 0.4 vs. 1.19 ± 0.1, *p* = 0.05). After surgery, tonsils pulsatility decreased significantly (323 ± 175 μL/cardiac cycle (CC) vs. 194 ± 130 μL/CC, *p* = 0.008) and subarachnoid CSF pulsatility at the foramen magnum increased significantly (201 ± 124 μL/CC vs. 363 ± 231 μL/CC, *p* = 0.02). After surgery, we found a decrease in venous flow amplitude (5578 ± 2469 mm^3^/s vs. 4576 ± 2084 mm^3^/s, *p* = 0.008) and venous correction factor (1.98 ± 0.3 vs. 1.20 ± 0.3 mm^3^/s, *p* = 0.004). Conclusions: Phase-contrast MRI could be a useful additional tool for postoperative evaluation and follow-up, and is complementary to morphological imaging.

## 1. Introduction

Chiari malformations (CMI) are defined radiologically and from a purely morphological point of view. They consist of ptosis of the cerebellar tonsils through the foramen magnum of greater than 3 [1] or 5 mm [2]. The foramen magnum is defined by the MacRae line. The radiological incidence of CMI is 0.77% of the global population [3] but not every patient presents symptoms. The clinical incidence was evaluated around 3.08:100,000 [4]. There is not one but several clinical presentations, consisting mainly of headaches, as well as visual (objective or subjective) and otoneurological symptoms, brainstem or cranial nerve symptoms, or medullary symptoms [5]. Most clinical signs are directly related to ptosis of the tonsils or the resulting complications, such as syringomyelia. In many cases, symptoms are not specific and the decision of whether to perform surgery or not can be extremely difficult. In addition, there are a number of differential diagnoses, such as idiopathic intracranial hypertension, which can radiologically mimic a CMI. Indeed, we observed ptosis of the cerebellar tonsils in 20.9% of patients with idiopathic intracranial hypertension [6] with non-specific headaches.

The treatment of symptomatic CMI is surgical [7] and consists of opening the foramen magnum bone, with or without duraplasty. In certain cases, the procedure includes a resection of the tonsil. CMI surgery has a significant complication rate. According to Klekamp et al., 21.8% of patients experience a complication, resulting in permanent morbidity for 3.2% [8]. In the aforementioned study, 73.6% of patients improved three months after surgery, but 14.3% showed neurological deterioration after five years [8]. Most recently, Ciaramitaro et al. proposed an international consensus regarding surgical indication for CMI and proposed strategies for diagnosis using specific scales for CMI [9].

CMI suggests by its name a malformation of a congenital nature. The pathophysiology of CMI is not clear but recent advances indicate that it is related to problems with intracranial pressure (ICP) and neurofluid dynamics.

Indeed, elevated cerebral spinal fluid (CSF) velocities at the level of the foramen magnum, associated with so-called compensatory pulsatility of the tonsils, have been described, but non-quantitatively. CMI decreases the subarachnoid space in the plane of the foramen magnum. Consequently, higher CSF velocities have been observed to preserve the flow rate at the level of the foramen magnum [10,11,12,13]. In cases of high disturbance of the flow rate, an increase in the pressure gradient and pulsatility of the neuro-axis were observed [14,15]. Non-invasive measurement of the pressure gradient has been performed [16]. The values for the non-invasive gradient measurements between the intracranial subarachnoid and cervical subarachnoid spaces correlated with the invasive measurements of the ICP amplitude. However, the non-invasive measurements of the pressure gradient for controls and patients were close.

ICP is defined by the pressure of the CSF. In 1926, ICP was conceptualized by Cushing as being subject to the Monro–Kellie doctrine [17]. This doctrine considers that all intracranial compartments must compensate each other in volume because their contents are incompressible. The contents consist of the CSF, the blood, and the brain parenchyma. Each change in volume in one compartment must be offset by a corresponding change in another. More recently, a new vision of the Monro–Kellie doctrine, called by its authors Monro–Kellie 2.0, has been proposed [18]. The authors proposed the importance of venous drainage, which mainly influences ICP. Impaired venous drainage and increased intracerebral resistance influence ICP. For example, in the situation of zero resistance to venous drainage, CSF oscillations in the cardiac cycle should be null, as would changes in the ICP amplitude.

The relevance of hydrodynamic analyses is a subject of debate. For some authors, tonsils pulsatility appears to be the main diagnostic marker of CMI [19] but there have been no quantitative analyses. Post-operatively, a decrease in CSF pulsatility through the aqueduct may be a marker of efficacy [20]. However, there is no consensus on this point, and for some, CSF velocities do not correlate with the clinical evolution [21], except for headaches (a decrease of >20% of the velocity is predictive of improvement) [12,22].

We propose that the appearance of a ptosis of the cerebellar tonsils and syringomyelia is linked to its own hemohydrodynamic mechanisms. We aimed to quantify CSF and cerebral blood flow to highlight how neurofluid is affected by CMI and its surgery.

## 2. Materials and Methods

### 2.1. Patients

We retrospectively included 21 adult patients with CMI who underwent pre- and postoperative MRI during the period from 2010 to 2017. The mean age of the subjects was 41 ± 16 (21–72) years.

The symptomatology was attributed by the neurosurgeon to be exclusively due to CMI. Patients with associated spina bifida (Chiari type 2) and those <18 years of age were excluded.

Patients were treated by either simple bone decompression or bone decompression associated with duraplasty. No patient was treated by tonsil excision. Inclusion was independent of the type of surgery performed.

Twelve patients presented with a syringomyelia cavity.

The surgical indication was determined based on symptoms related to the malformation, as well as on its radiological definition (each patient thus underwent encephalic MRI). We added phase-contrast MRI (PCMRI) sequences to the usual clinical morphology protocol (addition of 10 min of acquisition, on average).

### 2.2. MRI Acquisition

All patients underwent MRI as part of their conventional clinical assessment. MRI was performed distant from any lumbar puncture (more than three months). In addition to conventional clinical sequences, we added two PCMRI sequences.

**Figure 1:**
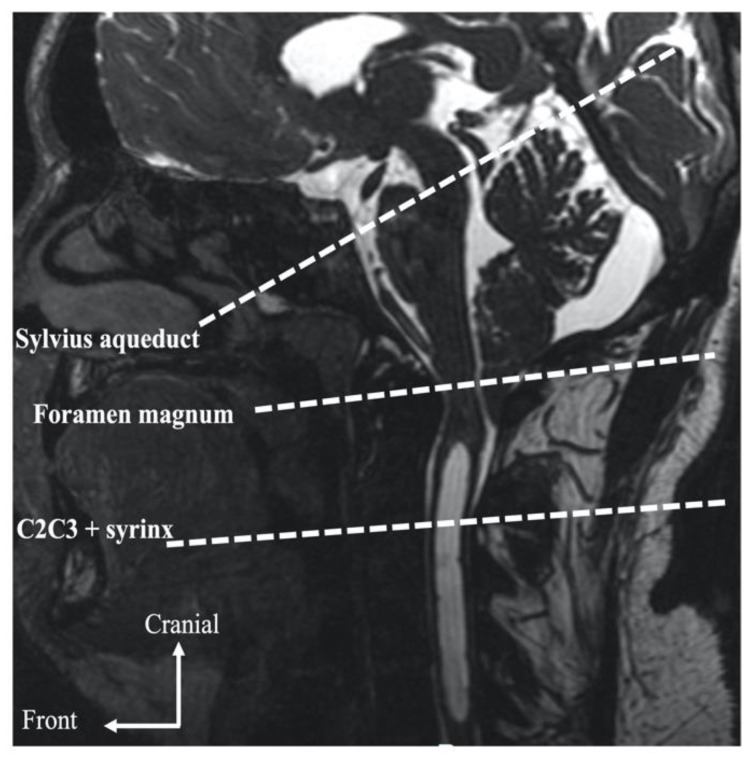
PCMRI acquisitions planes on sagittal T2 sequence.

Cerebral MRI was performed using a 3T scanner (GE Healthcare, Milwaukee, WI, USA). PCMRI was performed with the following parameters: the repetition time (TR) and echo time (TE) were selected with the minimum value depending on heart rate, field-of-view = 16 cm^2^, matrix = 256 mm^2^ or 128 × 256 mm^2^, slice thickness = 5 mm, flip angle = 30°, two excitations, and two views per segment. Peripheral cardiac synchronization was achieved with retrospective gating to reconstruct 32 temporal phases of one mean cardiac cycle calculated from all cardiac cycles during the acquisition. For CSF flow, we selected velocity encoding, (*VENC*) = 5 cm/s at the level of the cervical, foramen magnum, and syrinx (if present). Pulsatility of the tonsils were also quantified through the foramen magnum. The Venc was increased to 10 cm/s for the Sylvius aqueduct and if aliasing occurred. Internal carotid, vertebral, and jugular artery flow at the cervical level was quantified using *VENC* = 80 cm/s. CSF and blood flow acquisition slices were positioned perpendicular to the presumed direction of the flow (Figure 1).

### 2.3. Data Analysis

Data were analyzed using in-house software for the semi-automatic delineation of the blood vessels and CSF regions. Velocities and segmented areas were calculated through each of the 32 cardiac phases to produce the flow curves during the cardiac cycle. Stroke volumes were defined as the average of the cranio-caudal and caudo-cranial volumes displaced through the region of interest during the cardiac cycle [23]. Cerebral arterial blood flow (CBF_a_) was defined as the sum of the flow through the internal carotid and vertebral arteries.

Cerebral jugular blood flow (CBJF) was measured as the sum of the flow of the two internal jugular veins. As CBJF does not drain all the CBF itself (other peripherical veins are involved, such as epidurals, etc.) a venous correction factor was calculated equal to the mean (CBFa)/mean(CBJF). It was thus possible to provide a theorical *_corrected_*CBF_v_ flow curve = venous correction factor × CBJF flow. When this venous factor is equal to 1, the *_corrected_*CBF_v_ is equal to the CBF_v_. This corresponds to exclusive jugular venous drainage. When the correction factor is >1, it shows the magnitude of accessory drainage paths.

To evaluate how the cerebral blood volume increases during the cardiac cycle, the cerebral arteriovenous flow curve was calculated by subtraction of the CBF_a_ and *_corrected_*CBF_v_. The arteriovenous stroke volume was calculated as the CSF stroke volume.

The peak amplitude of the flow curves was calculated.

The complete postprocessing takes between 10 and 15 min to obtain all hemodynamic and hydrodynamic physiology of the patient.

Wilcoxon’s signed rank test for paired samples was used to compare the various hemodynamic and hydrodynamic parameters between the pre- and postoperative periods. Student’s *t*-test was used to compare hemodynamic parameters between patients with syringomyelia and those without. The threshold of significance was set to *p* = 0.05.

## 3. Results

### 3.1. Clinical Effectiveness of Surgery

Twelve patients underwent duraplasty enlargement and nine underwent bone decompression (Table 1). Twelve patients had initial syringomyelia (Table 1). The predominant preoperative symptomatology in the two groups was based on mainly functional cephalalgic symptomatology (14 patients, i.e., 67%) (Table 2).

All patients who underwent surgery stated that they felt better after surgery (100%). The symptoms remained less intense than preoperatively for three (25%), compatible with an optimal quality of life. Two patients with residual signs underwent extradural surgery and one underwent enlargement surgery.

Only three patients showed complete disappearance of their syringomyelic cavity (25%). Nine (75%) showed a persistent cavity but of much lower volume. For these patients, the syringomyelic cavity extended an average of 4.75 levels preoperatively and 2.5 postoperatively. Similarly, the mean maximum anteroposterior diameter of the cavities was 6.75 mm preoperatively and 3.5 mm postoperatively. On average, the syringomyelic cavities decreased by half after treatment. Only two patients in the syringomyelic population were operated by extradural approach.

No postoperative complications were described. One radiological complication without any clinical consequences, meningocele, was described. No surgical revision was reported.

### 3.2. Hemodynamics and Hydrodynamics in the Preoperative Period and Evolution after Surgery

No significant difference was found in the comparison of hemodynamic and hydrodynamic parameters between the groups of patients with syrinx and patients without syrinx (Table 3). Only the venous correction factor showed a very discrete significant difference (Table 3).

After surgery, there was a significant decrease in stroke volume measured at the level of the foramen magnum but not at the cervical level or at the level of the mesencephalic aqueduct (Table 4). We also observed a significant decrease in stroke volume measured at the level of the syrinx and stroke volume of the tonsils at the level of the foramen magnum (Table 4). Regarding the hemodynamic parameters, we observed a significant decrease in the amplitude of venous flow during the cardiac cycle and the venous correction factor (Table 4).

The analysis of the variations of the parameters after surgery did not reveal any difference between the two techniques (Table 5).

## 4. Discussion

The study of CSF dynamics and hemodynamics is possible by PCMRI for patients with CMI in 10 min complementary to the morphological sequences. Here, we show an alteration of both CSF dynamics and cerebral hemodynamics, particularly at the venous level.

### 4.1. Preoperative Hemodynamics and Hydrodynamics

CMI, by definition, decrease the surface of subarachnoid spaces at the cranio-cervical junction and thus decrease or block the local flow of CSF. Our current knowledge of the physiology of intracranial flow makes it possible to understand the crucial role of the flush of CSF towards the spinal subarachnoid spaces during systole [24,25,26,27]. Indeed, there is an increase in intracranial vascular volume at each cardiac systole [24,28]. Blood is not a “perfect fluid”; its viscosity is approximately three times higher than that of water and the vascular tree presents a high resistance to flow. This leads to an expansion in blood volume during the cardiac cycle, calculated as the SVvasc (stroke volume of arteriovenous in the cranium).

However, the cranial box is rigid and inextensible [25]. This increase in vascular volume is the origin of the “mobile compliance” of the CSF flush from the intracranial spaces to the compliant spinal subarachnoid spaces [24,26]. Nevertheless, although the Monro–Kellie doctrine states that intracranial volume is constant, it has been shown that this is not completely true during the cardiac cycle. CSF stroke volume in the spinal canal is smaller than arteriovenous stroke volume and the flow of CSF during the cardiac cycle does not completely balance arteriovenous flow [24,29]. Thus, ICP is not constant but shows P1, P2, and P3 amplitude peaks during the cardiac cycle [30].

In CMI, CSF intracranial “mobile compliance” is more or less restricted due to the decrease in the area of CSF circulation at the level of the foramen magnum. Pulsatility of the CSF measured at the level of the foramen magnum is strongly altered, as suggested by our results and those of previous studies [14,31]. Many CMI are asymptomatic due to the preservation of CSF oscillations, even if CSF velocities increase to maintain the same CSF volume oscillations in a smaller circulation area at the foramen magnum.

**Figure 2:**
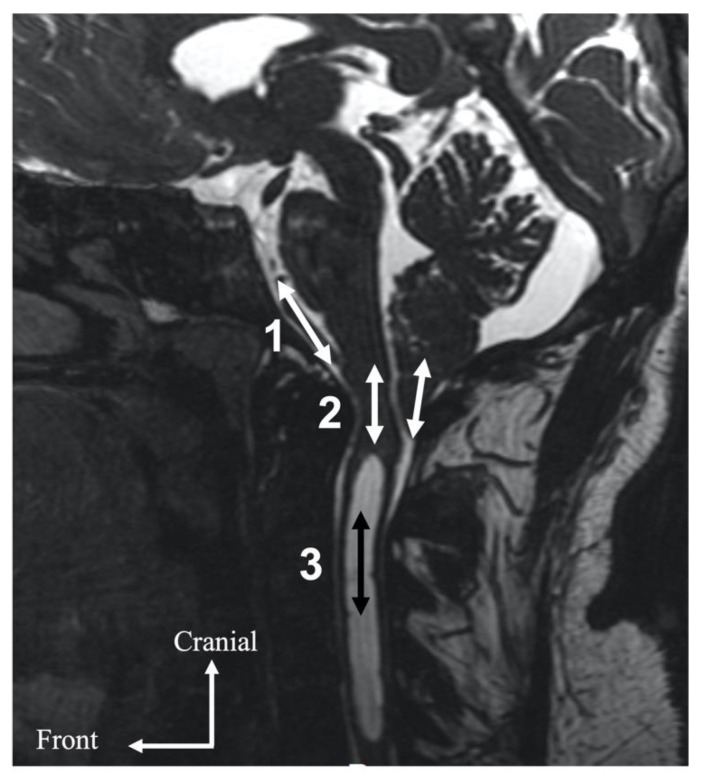
Local hydrodynamic localization of a Chiari malformation. The complete or partial alteration of CSF flow via the foramen magnum results in the transmission of CSF pulsatility from the intracranial subarachnoid spaces (1) to elongated marrow and cerebellar tonsils (2). This pulsatility is subsequently transmitted to the subarachnoid CSF or the syringomyelic cavity (3).

Paradoxically, we found no decrease in CSF dynamics measured at the upper cervical level in our population compared to a control population from the literature [24]. We explain this by the fact that pulsatility found at the cervical level does not directly come from the restricted CSF flow at the level of the foramen magnum but also from abnormal additional pulsatility of the medulla oblongata or cerebellar tonsils [14] through the foramen magnum. These parenchymal structures presented synchronized movement that was of the same order as that of the pulsatility amplitude of CSF measured at the cervical level. The “mobile compliance” required for the homeostasis of vascular intracranial flow via the medulla oblongata and cerebellar tonsils (Figure 2) help to mediate the regulation of ICP. These abnormal non-physiological movements of parenchymal tissues could lead to mechanical stress and become symptomatic.

There was no significant difference in the hemodynamic parameters with the presence of syringomyelia but patients with syringomyelia have a significantly higher venous correction factor. The venous correction factor reflects the participation of the jugular veins in cerebral blood drainage. When this factor is 1, drainage is exclusively jugular. When this factor tends towards 2, cerebral blood drainage is 50% jugular and 50% from alternative drainage routes. Therefore, patients with syringomyelia showed an important accessory venous drainage pathway that may be secondary to the greater alteration of craniospinal neurofluid interactions. This finding is borderline significant and needs to be explored by further specific analysis. Such a mechanism of the redistribution of venous drainage through small veins with a higher resistance to flow than the large jugular veins could produce barometric alterations. Such alterations in hemodynamics have also been shown to be present in idiopathic intracranial hypertension [28], in which CSF oscillations in the spinal canal are elevated. Moreover, ptosis of the cerebellar tonsils has been described in this same pathology without it being a CMI [6]. It is difficult to determine which phenomenon gives rise to the second.

Our results are not compared to a control population. This should be done in order to allow a better understanding of the physiopathological mechanisms. An analysis of this type would also allow us to observe if there are diagnostic markers complementary to the clinical and radiological morphological observations.

### 4.2. Evolution of Hemodynamic and Hydrodynamic Parameters after Surgery

The pre- and postoperative comparison of the craniospinal hemohydrodynamics of CMI showed the ventricular CSF flow measured in the aqueduct to be normal and unaffected by CMI. No patients showed hydrocephalus, which is not common in CMI.

The stroke volume of the tonsils was analyzed by a semi-automatic segmentation of the bulbar-medullary junction and the amygdala according to the same method as the CSF, this allows a quantification of the pulsatility of the tonsils during one cardiac cycle. The stroke volume of the tonsils, or pulsatility of tonsils, is the volume of tonsils displaced during one cardiac cycle. This displacement is in a craniocaudal direction during the systolic phase and in a caudocranial direction during the diastolic phase. Here, we show low CSF oscillations at the cranio-cervical junction and the existence of pulsatility of the tonsils, which decreased after surgery, associated with an increase in CSF volume pulsatility. Previous studies have shown higher CSF velocities at the cranio-cervical junction in Chiari populations than in a control population [13] and a decrease after surgery [13]. This can be easily explained by the fact that as CSF spaces decrease, the velocities increase, as well as the resistance to flow, which leads to a decrease in volume displacement for the same pressure. From our point of view, velocity alone is not the best parameter to evaluate the impact of CMI. It depends on the region of interest that is segmented and the anatomy of the foramen magnum, which can present multiple compartments. We prefer to consider the total CSF volume displaced trough the foramen magnum in the spinal canal, which must balance the intracranial blood volume expansion that varies from one person to another.

Moreover, duraplasty has been reported to allow for a greater decrease in pulsatility of the tonsils [15]. Nevertheless, this result may be affected by the time interval until the control MRI. Indeed, two patients in our population underwent repeated control MRI immediately postoperatively, and at three, six, and 12 months. One of the patients had surgery with bone decompression alone and the second with a complementary duraplasty. There was an immediate hydrodynamic impact of the surgery on the intradural patient. The patient treated with bone decompression alone showed progressive improvement in their CSF dynamics. In both cases there was a rapidly progressive improvement in symptoms. Therefore, there appears to be gradual enlargement of the large cistern postoperatively in the context of extradural surgery, making it possible to obtain the same results as intradural surgery but more gradually.

The pulsatility of the tonsils, not present in control populations, is a major marker of this pathology [19]. The decrease in the pulsatility of the tonsils may correlate with clinical improvement. In our study, 100% of patients showed improvement after surgery and a decrease in the pulsatility of the tonsils and medulla oblongata. Pulsatility of the tonsils would compensate for local hydrodynamic disturbances and alterations in CSF dynamics at the foramen magnum. Indeed, the restriction of the fluid spaces causes a decrease in the pulsatility of the CSF between the intracranial and spinal subarachnoid spaces. Such pulsatility is of major interest in the preservation of ICP [15,24]. One study calculated an index of the ICP gradient between intracranial and spinal compartments in CMI [16] and showed that the values of the non-invasive measurement of the pressure gradient correlate with those of the invasive measurement of ICP amplitude. Nevertheless, the values for the non-invasive measurements were close between the controls and patients. Our study shows a preoperative alteration of CSF dynamics at the craniospinal junction and the pulsatility of the tonsils. These findings, associated with data from the analysis of venous hemodynamics, confirm the existence of ICP disorders.

Hemodynamic analysis shows that the venous correction factor makes it possible to assess the venous drainage pathways (when equal to 1, drainage exclusively occurs through the jugulars, whereas when it is >1, some drainage occurs through accessory paths). Postoperatively, we observed a decrease in the correction factor that trended towards 1, showing that the involvement of jugular venous drainage is elevated in CMI. Decompression of the cranio-cervical junction made it possible to locally restore more physiological drainage. In addition, there was a decrease in venous flow amplitude after surgery. A number of studies have indeed shown a link between ICP disorders and this finding [26,32]. During decompression surgery of the craniocervical hinge, the large neck veins and epidural veins may be injured. This may lead to an alteration in their function and an iatrogenic decrease in the venous correction factor.

These results show the existence of a modification of the craniospinal hemodynamics and hydrodynamics after decompression surgery. There are cases of symptomatic recurrence which may lead to the question of a new surgery. The analysis of the neuro fluid dynamics may allow to observe if there is an alteration of the intracranial flows accompanying this recurrence of the symptomatology. This is an additional help to the decision of an often-difficult surgical indication.

## 5. Conclusions

Phase-contrast MRI is easy to use in clinical practice but requires quantitative post processing to be completely interpreted. In CMI, we observed an exclusive alteration in CSF dynamics at the foramen magnum plane accompanied by tonsils pulsatility. We observed a reduction in the amplitude of jugular blood outflow and modification of the cerebral venous drainage pathway. We also observed that surgery had a quantitative hemodynamic and hydrodynamic impact. Phase-contrast MRI could be a useful additional tool for postoperative evaluation and follow-up and is complementary to morphological imaging. These results could be used to reevaluate the classification of CMI by not only taking into account morphology but also information concerning neurofluid dynamics.

## Figures and Tables

**Table 1:** Population characteristics.

Population	Number of Patients
Sex ratio (M/F)	6/15
Age (years)	41 ± 16
Syrinx	12
Type of surgery	
Occipital craniectomy + C1 laminectomy Decompression + duraplasty	9 12

**Table 2:** Clinical evolution of patient symptoms.

	Preoperative	Postoperative
Headaches	14 (67%)	3 (14%)
Dizziness	5 (24%)	2 (10%)
Instability	8 (38%)	1 (5%)
Vestibular syndrome	2 (10%)	1 (5%)
Nystagmus	4 (19%)	1 (5%)
Ronchopathy	2 (10%)	0
Sleep apnea syndrome	3 (14%)	0
Paresthesia	6 (29%)	2 (10%)
Swallowing syndrome	3 (14%)	1 (5%)

**Table 3:** Preoperative hemodynamic and hydrodynamic parameters in syrinx and non-syrinx patients. CC: cardiac cycle, SVaqu: stroke volume of CSF in Sylvius aqueduct, SVforamen: stroke volume of CSF at the foramen magnum level, SVtonsils: stroke volume of tonsils, SVcerv: stroke volume of CSF at cervical level, SVvasc: stroke volume of arteriovenous system, AMPvenous: amplitude of venous flow curve, correction factor: factor of correction for venous flow curve—when it is close to 1, venous drainage is mainly due to the jugular veins, whereas, when it is >1, an accessory pathway is involved, SVsyrinx: stroke volume into the syringomyelia.

	Syringomyelia	No Syringomyelia	*p*
SVaqu (μL/CC)	24 ± 17	43 ± 25	0.13
SVforamen (μL/CC)	429 ± 317	176 ± 122	0.11
SVtonsils (μL/CC)	313 ± 197	332 ± 112	0.85
SVcerv (μL/CC)	458 ± 378	391 ± 155	0.62
SVvasc (μL/CC)	1649 ± 492	1689 ± 369	0.84
AMPvenous (mm^3^/s)	5201 ± 2391	6080 ± 2661	0.44
Correction factor	1.49 ± 0.4	1.19 ± 0.1	0.05

**Table 4:** Hemodynamic and hydrodynamic parameters evolution before and after surgery. CC: cardiac cycle, SVaqu: stroke volume of CSF in Sylvius aqueduct, SVforamen: stroke volume of CSF at the foramen magnum level, SVtonsils: stroke volume of tonsils (it is analyzed by a semi-automatic segmentation of the tonsils according to the same method as the CSF, this allows a quantification of the pulsatility of the tonsils during a cardiac cycle), SVcerv: stroke volume of CSF at cervical level, SVvasc: stroke volume of arteriovenous system, AMPvenous: amplitude of venous flow curve, correction factor: when >1, it shows that the jugular veins require an accessory venous pathway to drain all cerebral blood flow, SVsyrinx: stroke volume into the syringomyelia. Significant results are shown in bold.

	Preoperative	Postoperative	*p*
SVaqu (μL/CC)	39 ± 33	30 ± 26	0.36
**SVforamen** (**μL/CC**)	**201 ± 124**	**363 ± 231**	**0.02**
**SVtonsils** (**μL/CC**)	**323 ± 175**	**194 ± 130**	**0.008**
SVcerv (μL/CC)	434 ± 309	398 ± 241	0.73
SVvasc (μL/CC)	1661 ± 433	1490 ± 532	0.10
**AMPvenous** (**mm^3^/s**)	**5578 ± 2469**	**4576 ± 2084**	**0.008**
**Correction factor**	**1.38 ± 0.3**	**1.20 ± 0.3**	**0.04**
**SVsyrinx** (**μL/CC**)	**118 ± 86**	**26 ± 28**	**0.03**

**Table 5:** Comparison of hemodynamic and hydrodynamic parameter according to the type of surgery. All results are expressed as a percentage change. This is calculated as the ratio between the preoperative and postoperative values. A change of less than 100% indicates a decrease. A value greater than 100% indicates an increase. SVaqu: stroke volume of CSF in Sylvius aqueduct, SVforamen: stroke volume of CSF at the foramen magnum level, SVtonsils: stroke volume of tonsils (it is analyzed by a semi-automatic segmentation of the tonsils according to the same method as the CSF, this allows a quantification of the pulsatility of the tonsils during a cardiac cycle), SVcerv: stroke volume of CSF at cervical level, SVvasc: stroke volume of arteriovenous system, AMPvenous: amplitude of venous flow curve, SVsyrinx: stroke volume into the syringomyelia.

Variations in the Measurement of the Parameters Studied before and after Surgery (%)	Extradural Approach	Duraplasty	*p*
SVaqu	101 ± 24	71 ± 35	0.21
SVcerv	145 ± 99	106 ± 46	0.59
SVforamen	399 ± 236	453 ± 330	0.43
SVtonsils	35 ± 59	48 ± 55	0.52
SVvasc	89 ± 22	95 ± 29	0.26
AMPvenous	105 ± 18	97 ± 52	0.78
Correction factor	86 ± 11	65 ± 27	0.36
SVsyrinx	25 ± 35	20 ± 30	0.70

## Data Availability

Not applicable.
